# The impact of being of the female gender for household head on the prevalence of food insecurity in Ethiopia: a systematic-review and meta-analysis

**DOI:** 10.1186/s40985-020-00131-8

**Published:** 2020-06-05

**Authors:** Ayenew Negesse, Dube Jara, Habtamu Temesgen, Getenet Dessie, Temesgen Getaneh, Henok Mulugeta, Zeleke Abebaw, Tesfahun Taddege, Fasil Wagnew, Yilkal Negesse

**Affiliations:** 1 School of Human Nutrition, Food Science and Technology, Hawassa University, Hawassa, Ethiopia; 2 Department of Human Nutrition and Food Sciences, College of Health Science, Debre Markos University, Debre Markos, Ethiopia; 3 Department of Public Health, College of Health Science, Debre Markos University, Debre Markos, Ethiopia; 4 School of Public Health, College of Medicine and Health Science, Addis Ababa University, Addis Ababa, Ethiopia; 5 Department of Nursing, College of Health Science, Bahir Dar University, Bahir Dar, Ethiopia; 6 Department of Midwifery, College of Health Science, Debre Markos University, Debre Markos, Ethiopia; 7 Department of Nursing, College of Health Science, Debre Markos University, Debre Markos, Ethiopia; 8 Department of Health Informatics, University of Gondar, Gondar, Ethiopia; 9 Ethiopia Field Epidemiology and Laboratory Training Program (EFELTP), University of Gondar, Gondar, Ethiopia; 10 College of Medicine and Health Sciences, Mizan Tepi University, Mizan, Ethiopia

**Keywords:** Ethiopia, Female-headed households, Food insecurity, Meta-analysis, Systematic review

## Abstract

**Background:**

Ethiopia signed both for Millennium Development Goals (MDGs) previously and Sustainable Development Goals (SDGs) currently to improve food security through gender equality and empowerment of women by positioning them as household leader. However, there is no concrete evidence about the impact of being of the female gender for household head on the prevalence of food insecurity at the national level, the authors’ intention being to fill this gap.

**Methods:**

Preferred Reporting Items for Systematic Reviews and Meta-Analyses protocol (PRISMA-P) guideline was followed. All major databases such as PubMed/MEDLINE, WHOLIS, Cochrane Library, Embase, PsycINFO, ScienceDirect, Web of science, and reference lists were used to identify published articles, whereas shelves, author contact, Google, and Google Scholar were also searched to identify unpublished studies. Joanna Briggs Institute Meta-Analysis of Statistical Assessment and Review Instrument (JBI-MAStARI) was used for critical appraisal of studies. Meta-analysis was conducted using the STATA software version 14. The random effect model was used to estimate the pooled prevalence of food insecurity at 95% confidence level, while subgroup analysis and meta-regression were employed to identify the possible source of heterogeneity and the associated factors respectively. Moreover, Begg’s test was used to check publication bias.

**Results:**

A total of 143 articles were identified, of which 15 studies were included in the final model with a total sample size of 2084 female-headed households. The pooled estimate of food insecurity among female-headed households was 66.11% (95% confidence level (CL) 54.61, 77.60). Female-headed households had 1.94 (95% CL 1.26, 3.01) times the odds of developing food insecurity as compared with male-headed households in Ethiopia. However, considerable heterogeneity across studies was also exhibited (*I*^2^ = 92.5%, *p* value < 0.001).

**Conclusion:**

This review found that severity of food insecurity among female-headed households in Ethiopia was a more pronounced issue as compared with the general national estimate of food insecurity. Food insecurity among them was two-fold increased as compared with their men counterparts.

So that, the government of Ethiopia needs to outlook how cultural and social restriction of women’s involvement in every aspect of activity affects their level of household food security. Beyond this, previous success and current gap of food insecurity among female-headed households should be explored in future research to run in accordance with Sustainable Development Goals (SDGs) specially with goals 2 and 5.

## Introduction

The concept of food insecurity is explained by the quantity and quality of the available food, uncertainty about accessibility of food, and experiences of going hungry [1]. This concern is also experienced at the individual level with the issue of food consumption, allocation, and the physiological sensation of hunger [2].

Food insecurity can be measured by using the Food and Agriculture Organization (FAO) method for estimating calories available per capita at the national level, household income, and expenditure surveys; individual’s dietary intake; anthropometry; and experience-based food insecurity measurement scales [3]. However, the commonly used measurement tools of food insecurity in Ethiopia are Household Food Insecurity Access Scale (HFIAS) and Household Hunger Scale (HHS). The HFIAS was developed by the Food and Nutrition Technical Assistance Project, Academy for Educational Development 2007 tool [4], whereas the HHS was also developed by Food and Nutrition Technical Assistance III Project in collaboration with USAID and Tufts University [5].

According to the UN World Food Program report of 2017, 108 million people were faced with a severe form of food insecurity [6]. There were also 815 million people faced with hunger though there were enough food produced to feed the population globally [7].

The issue of household food insecurity is more pronounced in sub-Saharan African (SSA) countries, including Ethiopia. The dramatic change of global warming, slow development of the global economy, and regional conflicts have a tremendous role on food insecurity in SSA [8].

From SSA countries including Ethiopia, Food and Agriculture Organization reported that more than one in ten households were affected with food insecurity in the year 2016 [9], whereas in the year 2017, 5.6 million people needed emergency food support [10]. To combat the issue of food insecurity and other cross-cutting issues, Ethiopia adopted both the Millennium Development Goals (MDGs) in the past and Sustainable Development Goals (SDGs) currently [11, 12].

In spite of the challenges, the SDGs agendas of 2030 are now under implementation in Ethiopia. Lack of availability of data in terms of information revolution as part of women’s empowerment and food security in the developing countries in particular is a major constraint for monitoring and evaluation towards the progress of the activities and the ongoing outputs [13, 14]. The approaches of SDGs also usually lack an underlying theoretical foundation; so that, beyond the political commitment of each United Nation (UN) country on the SDGs, each strategic objective of SDGs should be re-designed through baseline theoretical or conceptual framework with inclusive hypothesis [15].

Once more, SDGs are long-term development agendas and have the potential to be exposed to unexpected negative outcomes [16].

Finally, being universal transformation agenda itself, people-centered and nature of comprehensiveness may also affect future successfulness of each strategic goal of SDGs [17]. Hence, special mechanisms tailored for equitable resource distribution along with alternative strategic plans and approaches across national and sub-regional level like in Ethiopia are warranted to meet the SDGs in 2030. Having all those challenges, Ethiopia as part of Africa is using gender equality and empowerment of women as a tool for combating hunger and achieving food security [18]. Women empowerment and gender equality is not only a tool and a human right, but also it is a necessary foundation for sustainable development for a given country too [19].

In fact, giving power to women to increase productivity and food security is also ascertained as a mechanism for gender equality and empowerment of women [20] though it is a more broad term in different contexts [21].

In Ethiopia, there are pocket and fragmented studies across regions that explore the relationship between being of the female gender for household head and food security [22–36]. However, the studies were inconclusive and there is no concrete scientific evidence established at national level. Therefore, the main objective of this study was to identify the impact of being of the female gender for household head on the prevalence of food insecurity in Ethiopia as part of SSA.

Hence, this review is helpful to identify the basic factors for such devastating issue and to undertake early measures for the further achievement of SDGs in particular of goals 2 and 5.

## Methods

### Search strategy

Initially databases were searched for both systematic review and meta-analysis to avoid duplications. First, DARE database (http://www.library.UCSF.edu) was explored in an attempt to confirm whether systematic review or meta-analysis exists and for availability of ongoing projects related to the topic. We also searched the two trial registries: ICTRP and Clinical Trials.Gov (searched December 2017). By using this method, it was confirmed that there was no review nor meta-analysis conducted linked to this topic.

All major databases such as PubMed/MEDLINE, WHOLIS, Cochrane Library, Embase, PsycINFO, ScienceDirect, Web of science, and reference lists were used to identify published articles, whereas shelves, author contact, Google, and Google Scholar were also used to identify unpublished studies. Our search for the published articles was restricted by the household head (female-headed households) and by country (studies conducted in Ethiopia only). All published and unpublished articles up to January 16, 2018, were included under this systematic review and meta-analysis. We followed the Preferred Reporting Items for Systematic Reviews and Meta-Analyses protocol (PRISMA-P) guideline for this meta-analysis [37].

The key terms used in building the search strategy were prevalence, magnitude, food security, food insecurity, female-headed household, and Ethiopia. The key terms were combined using Boolean operators to search the electronic databases. In addition, all fields and mesh terms were used while we used the advanced PubMed searching strategy.

### Eligibility criteria

#### Inclusion Criteria

This systematic review and meta-analysis considered all the studies which were conducted both in urban and rural parts of Ethiopia that reported numbers of male-headed households, female-headed households, and who experienced food insecurity. To estimate the pooled prevalence, only female-headed individuals were included, whereas to identify the association between being of the female gender for household head and food insecurity, articles which reported food insecurity both at female- and male-headed households were considered. This systematic review and meta-analysis were not restricted based on publication conditions, publication time, and study designs.

#### Exclusion Criteria

Based on the eligibility criteria, we read their titles and abstracts. If studies were relevant for our review, we examined the full texts. Those papers not fully accessed at the time of our search process were excluded after contact was attempted with the principal investigator through email at least two times. The reason for the exclusion of these articles is that we were unable to assess the quality of each article in the absence of their full texts. Moreover, studies which did not report our outcome of interest were excluded after reviewing their full texts. Studies which did not report food insecurity both at female- and male-headed households at the same time were also excluded from the final meta-analysis. Once more, studies with poor quality according to the settled criteria were also excluded from this systematic review and meta-analysis.

Quality assessment

The database search results were combined and duplicate articles were removed manually using Endnote (version X8). Joanna Briggs Institute Meta-Analysis of Statistics Assessment and Review Instrument (JBI-MAStARI) adapted for cross-sectional study design was used [38]. Four independent reviewers critically appraised each individual paper. Disagreements between those reviewers were solved by discussion. If not, a fifth reviewer was involved to resolve inconsistencies among them. Studies which scored 50% and above were included under this systematic review and meta-analysis.

### Data extraction

Data were extracted by the four authors using a standardized data extraction spread sheet. The data extraction spreadsheet was piloted on 5 randomly selected papers and modified accordingly. The data extraction sheet included study characteristics such as the following: (1) authors’ name, year, region, study or publication year, study design, study setting, and study population; (2) proportion of food insecurity; (3) information on the number of households who experienced either household food insecurity or security both from male- and female-headed households; and (4) studies’ quality score and sampling techniques.

### Outcome measurement

This study had one main outcome. It was household food insecurity among female-headed households. This was calculated as the number of female-headed households who were in food insecurity divided by total female-headed households and then multiplied by 100%.

### Data analysis and synthesis

The extracted data were entered into the computer using excel sheet and imported to STATA 14 for analysis. Evidence of publication bias and heterogeneity was assessed. Begg’s test with *p* value of less than 0.05 as a cutoff point to declare the presence of publication bias was considered [39]. Heterogeneity across studies was checked using Cochran Q statistic with the inverse variance (*I*^2^) value ranges from 30 to 60%, 50 to 90%, and 75 to 100% with moderate, substantial, and considerable heterogeneity across individual studies [40]. The forest plot was also used to visualize the presence of heterogeneity. A *p* value less than 0.05 was also used to declare the presence of heterogeneity across studies. Potential differences between the studies were explored by subgroup analysis and meta-regression. The findings were presented using forest plot with respective odds ratio and 95% confidence intervals via random effects meta-analysis (DerSimonian and Laired) model to estimate the summative effect about the impact of being of the female gender for household head on the prevalence of food insecurity. The impact of heterogeneity across studies on the meta-analysis was quantified by *I*^2^ statistic’s tau and a cutoff point of 50% was used to declare substantial heterogeneity. The effect size of categorical data was expressed using odds ratio.

## Results

### Search results

**Fig. 1:**
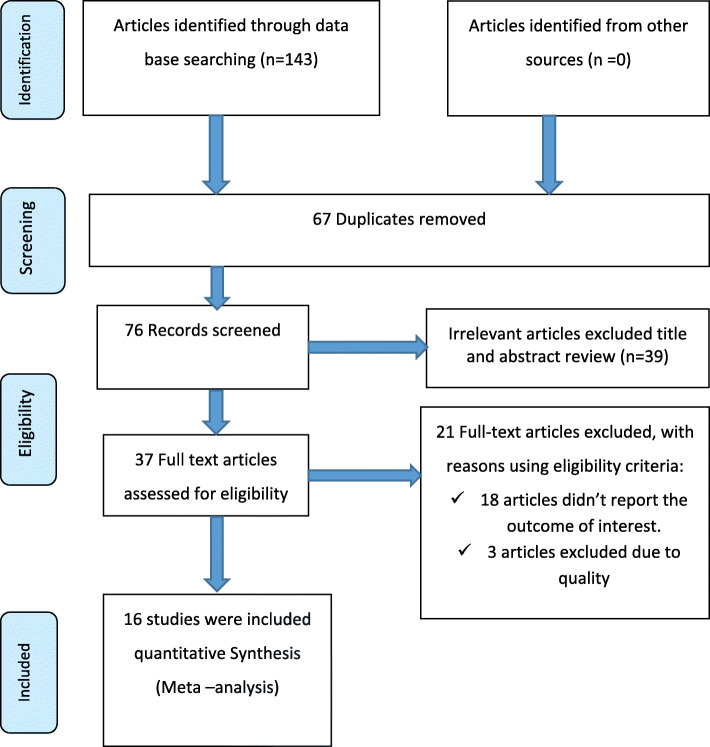
PRISMA-P flow diagram of the included studies to estimate the pooled impact of being of the female gender for household head on the prevalence of food insecurity in Ethiopia

A total of 143 studies were retrieved through our searches. Of these articles, 67 duplicated articles were excluded. From the remaining 76 articles, 39 articles were excluded after reading of titles and abstracts. Finally, 37 full-text articles were accessed and assessed for eligibility criteria. Based on the pre-defined criteria and after critical appraisal, only 15 articles were included in the final analysis (Fig. 1).

### Characteristics of the original articles

A total of 15 original studies were included under this systematic review and meta-analysis. All the included studies were cross sectional and had a pooled sample size of 2084 households (see Table 1). The minimum sample size (3 households) was used in a study conducted at Oromia regional state [27], whereas maximum sample size (581 households) corresponded to Tigray regional state [30].

**Table 1:** Characteristics of included studies to estimate the pooled prevalence among female-headed households in Ethiopia from 2007 up to 2017

Region	Author	Year	Study setting	Study design	Sample size	Type of literature	Quality score	Prevalence (95% CI)
Amhara	Motbainor et al. [24]	2016	community	Cross sectional	291	Article	8	55.67 (49.96, 61.38)
Amhara	Alem [22]	2016	Community	Cross sectional	49	Gray	6	77.55 (65.87, 89.23)
Amhara	Epherm [25]	2008	Community	Cross sectional	17	Gray	5	76.47 (56.31, 96.63)
Amhara	Motbainor et al. [24]	2016	community	Cross sectional	277	Article	8	66.43 (60.86, 71.99)
Amhara	Endale et al. [26]	2014	Community	Cross sectional	115	Article	8	92.17 (87.27, 97.08)
Oromia	Mequanent and Esubalew [28]	2015	Community	Cross sectional	23	Article	5	69.57 (50.76, 88.37)
Oromia	Beyene and Muche [23]	2010	Community	Cross sectional	24	Article	5	66.67 (47.83, 85.53)
Oromia	Mitiku et al. [29]	2012	Community	Cross sectional	27	Article	5	40.74 (22.21,59.27)
Oromia	Mequanent et al. [27]	2014	Community	Cross sectional	3	Article	5	66.67 (46.50, 86.83)
Tigray	Eyob [30]	2012	Community	Cross sectional	581	Gray	8	32.19 (28.39, 35.98)
Tigray	Tsegay [31]	2009	Community	Cross sectional	251	Gray	8	49.00 (42.82, 55.19)
SNNP	Shone et al. [32]	2017	Community	Cross sectional	143	Article	8	81.82 (75.50, 88.14)
SNNP	Leza and Kuma [34]	2015	Community	Cross sectional	100	Article	6	76.00 (67.63, 84.37)
SNNP	Tantu et al. [33]	2017	Community	Cross sectional	121	Article	7	65.29 (56.81, 73.77)
SNNP	Fikire and Bekele [36]	2014	Community	Cross sectional	41	Gray	5	75.61 (62.46, 88.75)
SNNP	Negussie and Alemayehu [35]	2013	Community	Cross sectional	21	Article	5	66.67 (46.50, 86.83)

Of the included studies, four studies were conducted in Amhara region [22, 24–26], four studies in Oromia region [23, 27–29], two studies conducted in Tigray region [30, 31], and five studies in SNNPR region [32–36]. Regarding their publication conditions, five studies [22, 25, 30, 31, 36] were gray literatures, whereas the remaining 11 studies [23, 24, 26–29, 32–35] were published in reputable journals. After peer and independent evaluations of individual studies, all studies scored in the range from 5 up to 8 out of 9 values (Table 1).

### Meta-analysis

**Fig. 2:**
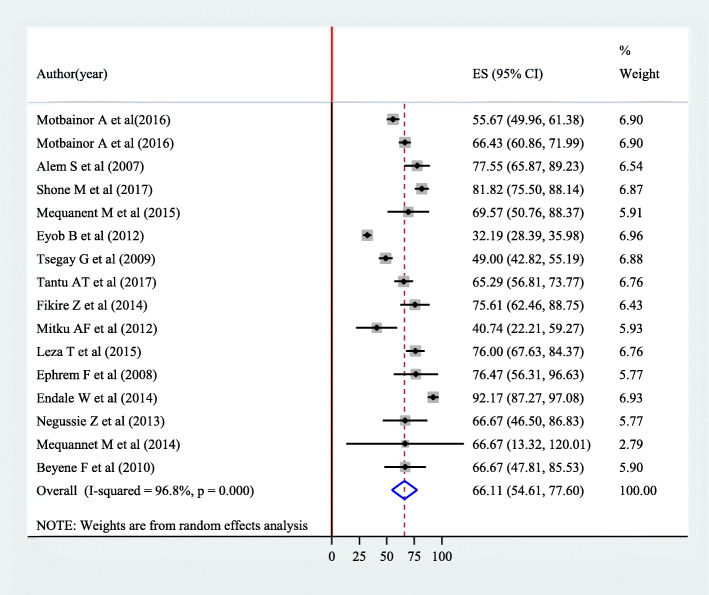
The pooled prevalence of food insecurity among female-headed households in Ethiopia

As indicated in the forest plot (Fig. 2), the pooled prevalence of food insecurity among female-headed households in Ethiopia was 66.11% (95% CI 54.61, 77.60). However, considerable heterogeneity was observed across studies (*I*^2^ = 96.8%, *p* value = 0.001). By considering this fact, random effect analysis was conducted.

### Sensitivity analysis

Sensitivity analysis was also done to identify outlier studies, but there was no any individual study which influenced the overall pooled estimate of this systematic review and meta-analysis.

### Subgroup analysis

Since this review is accounted for considerable heterogeneity, subgroup analysis based on region, publication condition, type of tool used to measure household food insecurity, and sampling technique were considered to identify the possible source of heterogeneity across studies (Table 2). However, the subgroup analysis result indicated that the source of heterogeneity was not due to regional, publication condition, and sampling technique differences. As reported in the subgroup analysis, the lowest and highest prevalence of food insecurity were 40.41% (95% CI 23.93, 56.89) and 74.18% (95% CI 67.13, 81.23) at Tigray and South Nations, Nationalities and Peoples of Ethiopia (SNNPs) respectively (Table 2).

**Table 2:** Subgroup analysis of the pooled prevalence of food insecurity among female-headed households in Ethiopia from 2007 up to 2017

Subgroup		Number of included studies	Prevalence (95% CI)	Heterogeneity statistics	*p* value	*I*^2^
By region	Amhara	5	73.48 (57.42, 89.54)	99.8	< 0.001	96.0
	Oromia	4	59.48 (43.93, 75.02)	5.65	0.130	46.9
	Tigray	2	40.41 (23.93, 56.89)	20.63	< 0.001	95.2
	SNNP	5	74.18 (67.13, 81.23)	10.16	0.038	60.6
By publication condition	Published	11	68.93 (59.30, 78.56)	123.06	< 0.001	91.9
	Unpublished	5	61.12 (42.34, 79.90)	102.42	< 0.001	96.1
By sampling method	Multi-stage	13	70.87 (62.46, 79.29)	123.15	< 0.001	90.3
	Survey	3	47.07 (31.05, 63.10)	29.99	< 0.001	93.3
By measurement tool	HFIAS	5	72.35 (58.34, 86.36)	109.31	< 0.001	96.3
	HHS	11	62.82 (49.19, 76.46)	171.83	< 0.001	94.2

Beyond the subgroup analysis, publication bias was checked using Begg’s test, though the value was find to be insignificant at *p* value of 0.753, which indicates there was no any study effect.

### Meta-regression

Beyond the subgroup analysis, meta-regression was also undertaken by considering both continuous and categorical data. Region where the study was conducted, sample size, year, publication condition, sampling technique, and studies conducted either of the two development goals and measurement tool for food insecurity were considered in the meta-regression. However, all the candidate variables in the meta-regression showed that heterogeneity in the prevalence of food insecurity was not associated with sample size variation, the tool used by authors to measure food insecurity, regional differences, type of sampling technique, publication year, and publication condition (Table 3).

**Table 3:** Meta-regression for the included studies to identify source of heterogeneity for the prevalence of food insecurity among female-headed rural households in Ethiopia from 2007 up to 2017

Variables	Characteristics	Coefficients	*p* value
Year	Publication year	− 0106513	0.501
Sample	Sample size	1.54988	0.782
Article	Un-published article	− 14.3361	0.640
Region	Amhara region	Reference	Reference
	Oromia	− 17.28004	0.694
	Tigray	− 34.76256	0.425
Sampling technique	Census survey	− 22.95167	0.470
	Multi-stage	Reference	Reference
Measurement tool	HFIAS	15.07132	0.674
	HFS	Reference	Reference
Development goals	SDGs	5.126678	0.574
	MDGs	Reference	Reference

Three studies [22, 31, 32] indicated that there was significant association between being of the female gender for household head and food insecurity in Ethiopia, whereas the remaining studies [23–30, 33–36] indicated that there was insignificant association between food insecurity and being of the female gender for household head. This indicated that the findings were not concrete and evidence for doing further systematic review and meta-analysis.

**Fig. 3:**
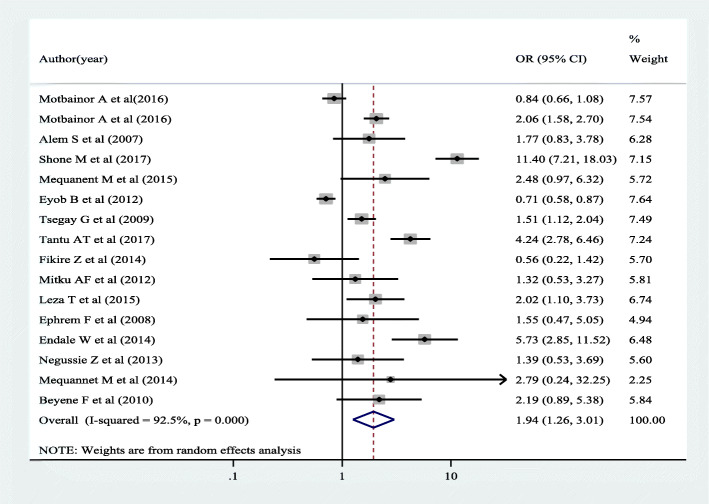
The association between being of the female gender for household head and food insecurity in Ethiopia

From this meta-analysis (Fig. 3), female-headed households had 1.94 (95% CL 1.26, 3.01) times the odds of developing food insecurity as compared with male-headed households. However, considerable heterogeneity across studies was also exhibited (*I*^2^ = 92.5 %, *p* value < 0.001).

## Discussion

This review focused on the prevalence of food insecurity among female-headed households and the effect of gender for household on food insecurity in Ethiopia from 2007 [22] up to 2017 [32, 33]. This review included studies which were done after the initiation of MDGs up to 2 years after Ethiopia signed the SDGs.

We found that two out of the three female-headed households in Ethiopia faced food insecurity. This issue was almost double as compared with male-headed rural households. This is incompatible with SDG 2 in which gender parity was settled as means of ending hunger, achieving food security and improved nutrition, and promoting sustainable agriculture for the year 2030 [41]. If gender preference on household head is not eliminated, the prevalence of food insecurity would increase by 12–17% [42]. It is also 39.11% greater than the regional overview of food insecurity across Northeast and North African countries conducted in the year 2014–2016 among the general population [43], and two times greater the South Sudan’s conducted on general population [44]. This discrepancy is more likely explained by geographic, social, cultural, and economic differences. Moreover, the current review considered only female-headed rural households. It is also six-fold greater than national estimates of food insecurity in Ethiopia [9]. This discrepancy may be due to study population differences and underestimated decision-making power of women [45].

Based on the subgroup analysis, Tigray was the most food secured region, whereas SNNPs were the least food secured region in Ethiopia. This can be explained by the well-connected community roads in Tigray region [46]. The relationship between food insecurity and public investment in road infrastructure was well established elsewhere [47]. Moreover, the human development index of Tigray region was relatively high as compared with other regions [48]. Human development index and food insecurity are interlinked to each other [49].

In comparison with male-headed households, female-headed households were more likely prone to food insecurity. Despite Ethiopia was one of the signatory countries to improve food security by implementing SDGs in particular of goals 2 and 5, the prevalence of food insecurity among female-headed households in Ethiopia was increased in the SDGs period as compared with the time of MDGs as showed in the subgroup analysis. So the previous success and the current flaws in food security among female-headed households should be explored in future research help to achieve all the SDGs in Ethiopia. This is supported by the systematic review and meta-analysis conducted both at global level and in other SSA countries [50–52]. This could be possibly explained by the fact that the decision-making power of women in the household may be undermined and women do not have the right to inherit their families capital [45] that coupled with their low income due to lower position in the labor force [52]. Beyond this, female-headed households also faced multiple challenges such as limited access to land ownership, market, and lack of agriculture extension services [53], and also because female-headed households cannot adopt new agricultural technologies, more prone to climatic changes [54, 55]. This also again can be explained by the fact that female-headed households specially from the rural areas may lack natural resources and information which are very important to mitigate food insecurity [56, 57]. However, no significant differences in food insecurity were found in a study conducted in Kenya, Uganda, and Tanzania [58] despite reasons were not provided. No significant differences of food insecurity among women and men were also found in a study conducted at Bangladesh. This can be explained because between both genders may have freedom to participate in the labor force without any social and cultural restrictions [59].

Our study has several strengths and limitations. Primarily, this systematic review and meta-analysis used internationally accepted tools for critical appraisal system for quality assessment of individual studies. Secondly, it included both published and unpublished studies. Since there were no original studies reported from some regional states of Ethiopia, this systematic review and meta-analysis may not represent all female-headed households in Ethiopia. This review included only cross-sectional studies, in which longitudinal studies are strong enough to indicate the true cause-effect relationship between being of the female gender as household head and food security [60].

Time differences between individual studies, variation of tools to measure outcome variable, sample size, nature of data collected, and geographic location may affect the outcome. However, appropriate statistical model (random effect model) along with best statistical estimation approaches such as subgroup analysis and meta-regression were considered to narrow this limitation.

Once more, this systematic review and meta-analysis also appreciated that the nature of female individuals in their leadership style to cope with food insecurity may also affect the overall pooled estimate of food insecurity. Hence, this is also the interest of the authors to warrant further exploration about the relationship between leadership style among female-headed households and food security outcomes.

## Conclusion

This systematic review and meta-analysis found that the prevalence of food insecurity among female-headed households was higher than the national estimate of household food insecurity and a significant association was found with gender as household head. Therefore, the government of Ethiopia should work to address cultural and social restrictions of women’s involvement as if affects their level of household food security. As more than half of the Ethiopian population is female, previous success and current flaws in food security among female-headed households should be explored in future research immediately to run in accordance with the SDGs specially with goals 2 and 5. Beyond this, it is also better to focus on the expansion of community roads and on activities which may increase human development index of the country in order to be contemplated with food security.

## Data Availability

The data sets analyzed during the current study are available from the corresponding author upon reasonable request.

## Abbreviations

FAO: Food and Agriculture Organization
HFIAS: Household Food Insecurity Access Scale
HHS: Household Hunger Scale
MDGs: Millennium Development Goals
PRISMA-P: Preferred Reporting Items for Systematic Reviews and Meta-Analyses protocols
SDGs: Sustainable Development Goals
SNNPs: South Nations Nationalities and Peoples
SSA: Sub-Saharan Africa
